# ^177^Lu-octreotate therapy for neuroendocrine tumours is enhanced by Hsp90 inhibition

**DOI:** 10.1530/ERC-18-0509

**Published:** 2019-02-07

**Authors:** Tobias Hofving, Viktor Sandblom, Yvonne Arvidsson, Emman Shubbar, Gülay Altiparmak, John Swanpalmer, Bilal Almobarak, Anna-Karin Elf, Viktor Johanson, Erik Elias, Erik Kristiansson, Eva Forssell-Aronsson, Ola Nilsson

**Affiliations:** 1 Department of Laboratory Medicine, Institute of Biomedicine, Sahlgrenska Cancer Center, Sahlgrenska Academy at University of Gothenburg, Gothenburg, Sweden; 2 Department of Radiation Physics, Institute of Clinical Sciences, Sahlgrenska Cancer Center, Sahlgrenska Academy at University of Gothenburg, Gothenburg, Sweden; 3 Department of Medical Physics and Biomedical Engineering, Sahlgrenska University Hospital, Gothenburg, Sweden; 4 Department of Surgery, Institute of Clinical Sciences, Sahlgrenska Academy at University of Gothenburg, Gothenburg, Sweden; 5 Department of Mathematical Sciences, Chalmers University of Technology, Gothenburg, Sweden

**Keywords:** neuroendocrine tumours, ^177^Lu-octreotate therapy, peptide receptor radionuclide therapy, Hsp90 inhibitor, ganetespib

## Abstract

^177^Lu-octreotate is an FDA-approved radionuclide therapy for patients with gastroenteropancreatic neuroendocrine tumours (NETs) expressing somatostatin receptors. The ^177^Lu-octreotate therapy has shown promising results in clinical trials by prolonging progression-free survival, but complete responses are still uncommon. The aim of this study was to improve the ^177^Lu-octreotate therapy by means of combination therapy. To identify radiosensitising inhibitors, two cell lines, GOT1 and P-STS, derived from small intestinal neuroendocrine tumours (SINETs), were screened with 1224 inhibitors alone or in combination with external radiation. The screening revealed that inhibitors of Hsp90 can potentiate the tumour cell-killing effect of radiation in a synergistic fashion (GOT1; false discovery rate <3.2 × 10^−11^). The potential for Hsp90 inhibitor ganetespib to enhance the anti-tumour effect of ^177^Lu-octreotate in an *in vivo* setting was studied in the somatostatin receptor-expressing GOT1 xenograft model. The combination led to a larger decrease in tumour volume relative to monotherapies and the tumour-reducing effect was shown to be synergistic. Using patient-derived tumour cells from eight metastatic SINETs, we could show that ganetespib enhanced the effect of ^177^Lu-octreotate therapy for all investigated patient tumours. Levels of Hsp90 protein expression were evaluated in 767 SINETs from 379 patients. We found that Hsp90 expression was upregulated in tumour cells relative to tumour stroma in the vast majority of SINETs. We conclude that Hsp90 inhibitors enhance the tumour-killing effect of ^177^Lu-octreotate therapy synergistically in SINET tumour models and suggest that this potentially promising combination should be further evaluated.

## Introduction

The ^177^Lu-[DOTA^0^,  Tyr^3^]-octreotate therapy, or ^177^Lu-octreotate therapy, is targeted against well-differentiated neuroendocrine tumours expressing somatostatin receptors. Following a promising retrospective study (Kwekkeboom *et al.* 2008, Brabander *et al.* 2017), it was recently shown in a phase 3 trial that ^177^Lu-octreotate markedly increased progression-free survival (65.2% vs 10.8% after 20 months) and significantly improved response rates (18% vs 3% after 20 months) in patients with small intestinal neuroendocrine tumours (SINETs), compared with the best standard of care (Strosberg *et al.* 2017). This has led to an FDA approval of ^177^Lu-octreotate therapy for gastroenteropancreatic NETs and its inclusion in treatment guidelines (Hicks *et al.* 2017). However, although response rates were improved, partial and complete responses (17% and 1% respectively) after ^177^Lu-octreotate therapy were still limited, emphasising the need to further optimise ^177^Lu-octreotate therapy. It has been shown in a human SINET xenograft model that administration of ^177^Lu-octreotate at high enough doses may result in complete tumour remission (Kölby *et al.* 2005). Increasing the dose may also have beneficial effects in the clinical setting, but could also give increased adverse effects. The most commonly reported severe adverse effects from ^177^Lu-octreotate therapy include renal failure, haematological toxicity and gastrointestinal disorders (Bergsma *et al.* 2016, Brabander *et al.* 2017, 2018). An alternative to increasing the treatment dose would be to use a combination therapy which improves the beneficial effect of ^177^Lu-octreotate without increasing the adverse effects (Fitzgerald *et al.* 2006). Attempts to combine ^177^Lu-octreotate with compounds that can enhance the therapeutic efficacy have been performed in preclinical studies (Elf *et al.* 2017, Spetz *et al.* 2017) and clinical studies (Claringbold & Turner 2015, 2016, Kashyap *et al.* 2015), with varying effect and without reported synergistic effects. Large-scale screening for candidate inhibitors that can enhance ^177^Lu-octreotate therapy and that could be used for combination therapy has not yet been performed.

In the present study, we performed a synergy screening to identify inhibitors that could enhance ^177^Lu-octreotate therapy. We found that the heat shock protein inhibitor ganetespib enhanced the tumour-killing efficacy of ^177^Lu-octreotate therapy in a synergistic manner, as demonstrated in SINET models *in vitro*, *in vivo* and *ex vivo*.

## Materials and methods

### Cell lines and patient-derived tumour cells

The GOT1 cell line was established from a liver metastasis of a midgut carcinoid (Kölby *et al.* 2001) and was cultured in RPMI1640 supplemented with 10% foetal bovine serum (FBS), 5 μg/mL insulin and 5 μg/mL transferrin. The P-STS cell line was a gift from Professor R Pfragner. It was established from the primary tumour, described as a grade 3 NET located in the terminal ileum (Pfragner *et al.* 2009), and was cultured in M199:Ham’s F12 (1:1) supplemented with 10% FBS. The cell lines were regularly tested for *Mycoplasma* species as described by van Kuppeveld *et al*. (van Kuppeveld *et al.* 1994) at a Swedac SS-EN ISO 15189 accredited laboratory (Sahlgrenska University Hospital, Gothenburg, Sweden). The identity of the cell lines was validated by STR analysis (Hofving *et al.* 2018).

Patient-derived tumour cells were established from biopsies of metastatic SINETs collected at the time of surgery, and prepared as previously described (Arvidsson *et al.* 2010). Clinical and histopathological data on patients and tumours are given in Table 1. The purity of primary cell cultures was assessed by light microscopy using haematoxylin and eosin-stained sections from cell blocks and was shown to be ≥95%. All patient-derived tumour cells were treated 24 h after establishment and kept in RPMI1640 supplemented with 4% FBS during experiments. The medium of all cell lines and patient-derived tumour cells also contained 100 IU/mL penicillin and 100 μg/mL streptomycin.

**Table 1:** Clinicopathological characteristics of patients with small intestinal neuroendocrine tumours used to evaluate ^177^Lu-octreotate synergy.

Patient #	Age at surgery	Gender	Tumour site^a^	Grade (WHO 2010)^a^	KI67 (%)^a^	Stage (TNM 7th edn)	SSTR2 score (0–3)	HSP90 score (high/low)
1	81	M	LN	G1	0.3	IV	2	High
2	76	M	LN	G1	0.6	III	3	High
3	59	F	LN	G1	1.1	IV	2	High
4	68	M	LN	G2	3.3	III	3	Low
5	40	M	L	G1	0.3	IV	3	Low
6	61	M	L	G1	0.4	IV	2	High
7	67	M	L	G2	2.3	IV	2	High
8	71	F	L	G2	2.4	IV	3	High

^a^Data for the specific tumour from which primary cells were derived.

F, female; L, liver metastasis; LN, lymph node metastasis; M, male.

### Pharmaceuticals and external radiation

The screening library consisted of 1224 inhibitors (L1100, Selleckchem). The library was batched in 96-well plates and stored at −80°C. The plates were subjected to no more than five freeze-thaw cycles. For *in vitro* experiments, ganetespib (Selleckchem) was aliquoted in DMSO and for *in vivo* experiments prepared in 5% DMSO and 45% PEG in ddH_2_O by applying a low amount of heat until dissolved, and was stored at −20°C.

Radiolabelling of [DOTA^0^,  Tyr^3^]-octreotate with ^177^Lu and subsequent thin-layer chromatography quality control was performed as previously described (Dalmo *et al.* 2017). The proportion of peptide-bound ^177^Lu was >99%. Each syringe was measured before and after administration of the radiopharmaceutical with a well-type ionisation chamber (CRC-15R, Capintec).

External radiation was given using a Varian TrueBeam linear accelerator with 6-MV nominal photon energy and a dose rate of 8.2 Gy/min absorbed dose to water. During irradiation, cell culture plates were held in a 220 × 220 × 90 mm^3^ polystyrene phantom. The height of the media in each well was 2 mm and the thickness of the cell layer was approximately 20 µm. The cell layer was positioned at a height corresponding to the distance for the dose maximum of the radiation beam. Irradiation was given at a 180° gantry angle (upwards irradiation), the source surface distance was 900 mm. The radiation field at the isocenter (1000 mm) was 200 × 200 mm^2^.

### In vitro cell experiments

For all experiments, 50,000 cells were seeded onto black non-optical 96-well cell culture plates (Nunc, Thermo Fisher Scientific) and 24 h was allowed for cell attachment before the start of the experiment. At the end of all experiments, the cell viability was estimated using alamarBlue (Thermo Fisher Scientific), a fluorescence-based assay for measurement of the reducing capacity of metabolically active cells. All assay plates were incubated for 6 h at 37°C, and were then analysed with a fluorescence plate reader (Wallac 1420, PerkinElmer; ex. 560 nm and em. 640 nm).

For screening experiment, the inhibitor library was added to 96-well culture plates, with each plate also including internal DMSO controls (*N* = 8). In addition, each experiment included one cell-free plate for background subtraction (*N* = 96) and one DMSO-treated plate as vehicle control (*N* = 96). The screening library was added to a final concentration of 1 µM. After adding the inhibitor library, combination-treated cells were subjected to 3 Gy external radiation within 30 min. Experiments were repeated two or three times for inhibitors only, for radiation only and for combination treatment. Screening experiments were ended after 48 h. For cell line dose-response experiments with ganetespib, GOT1 and P-STS were treated with ganetespib at indicated doses for 72 h before plates were analysed. Each dose included four replicates and the experiment repeated three times. For experiment with patient-derived tumour cells, first-passage cell cultures were generated from eight patients. The cells were treated with radiation (0 Gy, 7.5 Gy, or 10 Gy), ganetespib (0, 0.1, 1, or 10 µM) or both treatments combined (all dose combinations). For each patient and treatment the experiment included 12 replicates and was performed once due to the limited cell numbers. Cell plates were analysed 72 h after the start of the experiment.

### Tumour xenograft model and animal handling

GOT1 tissue was transplanted subcutaneously to BALB/c nude mice (Janvier Labs, Le Genest-Saint-Isle, France) as previously described (Kölby *et al.* 2001, Dalmo *et al.* 2017). The tumour response was evaluated in mice treated with a single intravenous tail vein injection of vehicle (*N* = 6), 15 MBq ^177^Lu-octreotate (*N* = 5), 50 mg/kg ganetespib (*N* = 5) or a combination of both treatments (*N* = 5). These amounts, and the single-administration design, were chosen in order to obtain a moderate but clearly measurable treatment effect of each monotherapy. Mouse tumours were measured twice weekly with slide callipers. Growth inhibition was calculated as the relative tumour volume compared to vehicle-treated mice at a given time point. Tumour regression was calculated as the relative tumour volume compared to treatment start. Water and autoclaved food were available *ad libitum*. After 14 days the experiment was ended and mice sacrificed by intraperitoneal injection of 60 mg/mL pentobarbital (Pentobarbitalnatrium vet., Apotek Produktion & Laboratorier), followed by cardiac puncture.

### Chromogranin A assay

When mice were sacrificed at the end point of the experiment, plasma was collected and human chromogranin A was quantified using ELISA according to the manufacturer’s instructions (ab196271, Abcam). All samples and chromogranin A standards were analysed using two technical replicates.

### Tissue microarray

The tissue microarray was generated from patients who underwent surgery for SINETs at Sahlgrenska University Hospital in the years 1986 to 2013. Details of the construction of the tissue microarray have been described previously (Arvidsson *et al.* 2018).

### Immunohistochemistry

Immunohistochemistry was performed on xenografted tumours, patient-derived tumour cells, patient tumour tissue and tissue microarrays. Tumours xenografted to mice were immediately excised after the mouse was sacrificed, and fixed in 4% buffered formaldehyde and then embedded in paraffin. Patient-derived tumour cells were fixed in methanol. Cell blocks from patient-derived tumour cells were created using a Cellient automated cell block system (Hologic). Tissue blocks from patient tumours were obtained from Sahlgrenska University Hospital. These blocks were prepared for routine clinical histopathology. Tissue microarrays were generated as described above.

Sections (3–4 μm) from paraffin blocks were placed on glass slides and treated in Dako PT-Link using EnVision FLEX Target Retrieval Solution (high pH). The following primary antibodies were used: anti-chromogranin A (PHE5, Chemicon and EP1030Y, Abcam), anti-synaptophysin (DAK-SYNAP, Dako and SP11, Abcam), anti-5HT (H209, Dako and LS-B7118, LSBio), anti-pan-cytokeratin (AE1/AE3, Dako), anti-SSTR2A (UMB-1, Abcam), anti-Ki67 (MIB-1, Dako) and anti-Hsp90 (EPR3953, Abcam). Immunohistochemical staining was performed in a Dako Autostainer Link using EnVision FLEX according to the manufacturer’s instructions (DakoCytomation). EnVision FLEX+ (LINKER) rabbit or mouse was used for all stainings except anti-chromogranin A (PHE5) and anti-pan-cytokeratin. Positive and negative controls were included in each run. Tumour regression was evaluated by board-certified pathologist (O.N.) and scored according to Becker *et al.* (2003) into four categories: ‘1a’ (no tumour), ‘1b’ (<10% remaining tumour), ‘2’ (10−50% remaining tumour) and ‘3’ (>50% remaining tumour). Scoring of Hsp90 expression in tumour cells vs stroma was evaluated in 701 SINETs from 369 patients and was based on the staining intensity of tumour cells relative to surrounding stromal cells and was categorised as *lower*, *equal* or *higher*. The overall Hsp90 expression was evaluated in 767 SINETs from 379 patients and based on the staining intensity of tumour cells scored as ‘1’ or ‘2’. The Ki67 index was calculated by manually counting the percentage of labelled tumour cell nuclei on printed images (Reid *et al.* 2015).

### Ethics approval

We obtained approval from Regional Ethical Review Boards in Gothenburg, Sweden, for the use of clinical materials for research purposes and for all animal procedures. Consent has been obtained from each patient after full explanation of the purpose and nature of all procedures used.

### Data analysis

For each cell line, inhibitor fluorescence intensities generated by the cell viability assay were log-transformed and normalised to a common scale. For each inhibitor, the interaction between radiation and inhibition was analysed using a two-way ANOVA with an interaction term. Significance was calculated based on the size of the interaction term. The false discovery rates (FDRs) were calculated using the Benjamini–Hochberg algorithm (Benjamini & Hochberg 1995). Overrepresentation of inhibitor classes among significant and non-significant inhibitors (*P* < 0.05) was assessed using Fisher’s exact test. The FDRs for the overrepresented inhibitor classes were calculated as above. The analysis of the longitudinal *in vivo* experiment was done based on the log-transformed relative tumour size of xenografted tumours. A linear mixed model was fitted to tumour volume measurements for each treatment (vehicle, external radiation, Hsp90 inhibition and combination treatment) with a random slope parameter modelling the growth rate. Synergy between treatments was assessed by comparing the effect size (slope) of the combined treatment with the sum of the effect sizes from the individual treatments. Significance was calculated using a Z-test with effect sizes and standard errors estimated by the linear mixed models. Any *P* value less than 0.05 was considered statistically significant. Comparisons of regression score (Becker score) between treatment groups was done using the Mann–Whitney nonparametric test. The analysis of the cell viability data from the experiments with patient-derived tumour cells was done as follows. Technical replicates were averaged and relative intensities, in comparison to the control, were calculated. For each radiation level (0, 7.5 and 10 Gy), a linear model was used to estimate the trend in relation to the log-transformed concentration of ganetespib. The difference in slope between radiation and vehicle control was used to assess synergy. Any *P* value less than 0.05 was considered statistically significant. Survival analysis, testing the difference in overall survival from time of surgery between high and low expression of Hsp90 was performed using Kaplan–Meier estimator with log-rank test. For patients with multiple tumours (more than one tumour site) only one tumour was included with the following priority: primary tumour, lymph node metastasis and hepatic metastasis. The survival analysis using the full patient cohort consisted of 312 patients and the ^177^Lu-octreotate-treated cohort consisted of 43 patients.

## Results

### Inhibitors of heat shock protein 90 synergised with ionising radiation to kill neuroendocrine tumour cells

**Figure 1:**
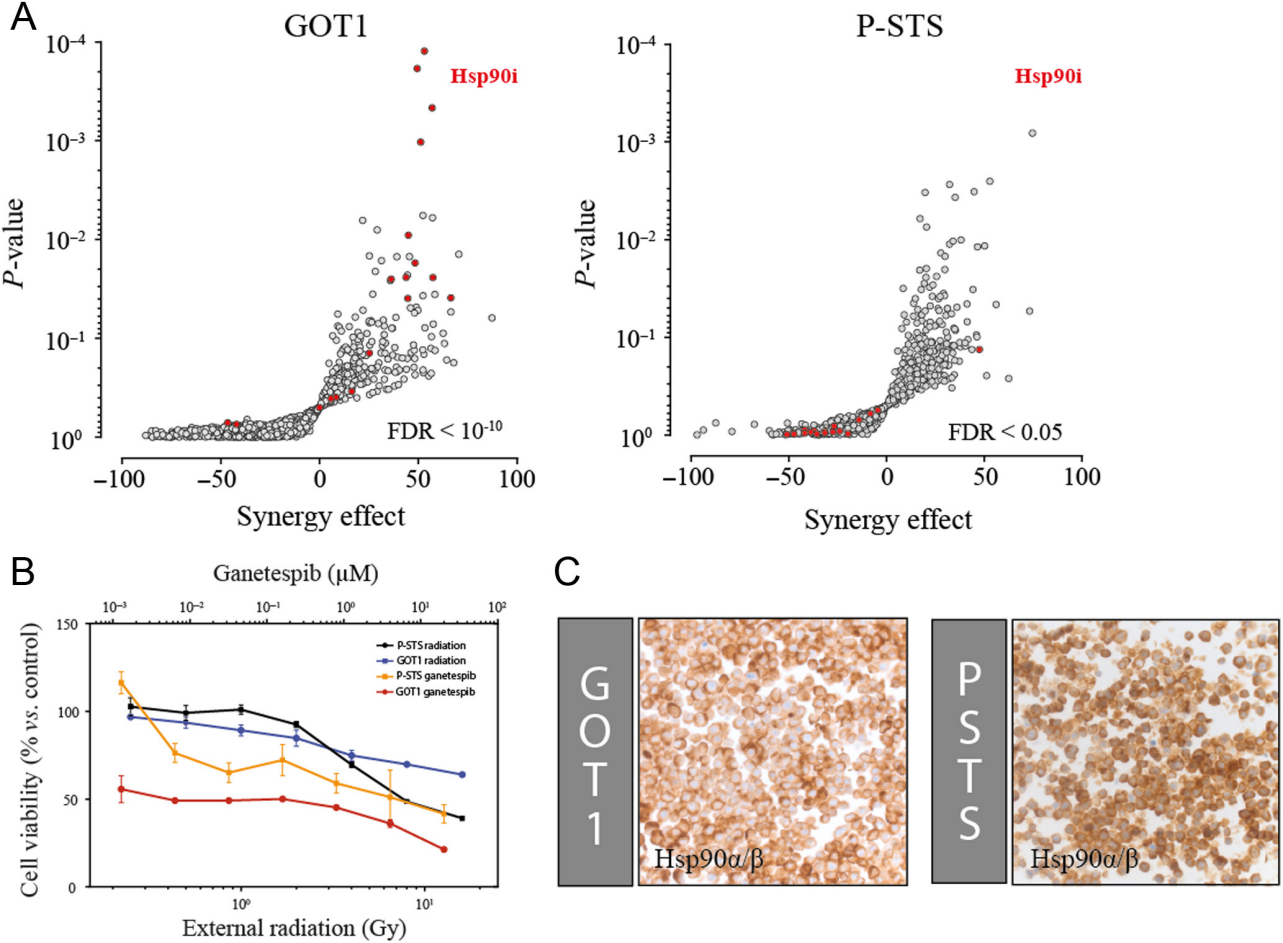
Effect of Hsp90 inhibition in combination with external radiation on GOT1 and P-STS. GOT1 and P-STS cells were treated with 1224 inhibitors alone and in combination with external radiation. (A) Hsp90 inhibitors (red dots) were overrepresented among inhibitors that significantly synergised with external radiation to decrease the viability of GOT1 cells, but not P-STS cells. (B) Dose-response curves of external radiation and ganetespib on the viability of the GOT1 and P-STS cell lines. Dots represent mean and error bars show standard deviation. (C) Immunohistochemical staining of SINET-derived cell lines confirmed the expression of Hsp90α/β.

In an attempt to identify inhibitors that might increase the potency of ^177^Lu-octreotate therapy in a synergistic manner, we screened cell lines GOT1 and P-STS using an inhibitor library consisting of 1224 inhibitors, both as single agents and in combination with external radiation. We were able to identify 28 and 37 inhibitors, respectively, in screenings of GOT1 and P-STS cells that significantly synergised with radiation (*P* < 0.05) in killing tumour cells *in vitro* (Supplementary Table 1, see section on supplementary data given at the end of this article). I-BET151, a BET bromodomain inhibitor (Dawson *et al.* 2011), showed significant synergistic effects in both cell lines. Using a group analysis of inhibitor classes, Hsp90 inhibitors were found to be strongly overrepresented among significant synergistic inhibitors in the GOT1 cell line (FDR = 3.2 × 10^−11^), with 11 out of 18 inhibitors having a significant synergistic effect (Fig. 1A). None of the Hspi showed however synergistic effects in the P-STS cell line (0/18). Apart from Hsp90i, no other inhibitor class was significantly overrepresented (FDR <0.05) among synergistic inhibitors in either cell line. P-STS was more sensitive to external radiation compared to GOT1 at IC50 (IC_50_ = 7.2 Gy vs not reached) and GOT1 was more sensitive to ganetespib compared to P-STS at all tested concentrations (Fig. 1B). The expression of Hsp90α/β protein in both cell lines was confirmed by immunohistochemical staining (Fig. 1C).

### ^177^Lu-octreotate therapy was enhanced by heat shock protein inhibition in the GOT1 xenograft

First-generation Hsp90 inhibitors have been shown to sensitise cancer cells to radiation (Dote *et al.* 2006, Lauber *et al.* 2015, Wang *et al.* 2016). In our screening experiments, we observed that Hsp90i synergised with external radiation in the SINET-derived cell line GOT1. We hypothesised that ganetespib, an Hsp90 inhibitor with a manageable side effect profile (Socinski *et al.* 2013), would also enhance the radionuclide ^177^Lu-octreotate therapy. To study this effect *in vivo*, we used the GOT1 xenograft model, which is advantageous for studies of peptide receptor radiotherapy due to its expression of somatostatin receptor subtype 2 (SSTR2) (Kölby *et al.* 2005, Swärd *et al.* 2008, Hofving *et al.* 2018). The tumour response was evaluated in mice treated with single intravenous injections of vehicle, ^177^Lu-octreotate, ganetespib or a combination of both treatments.

**Figure 2:**
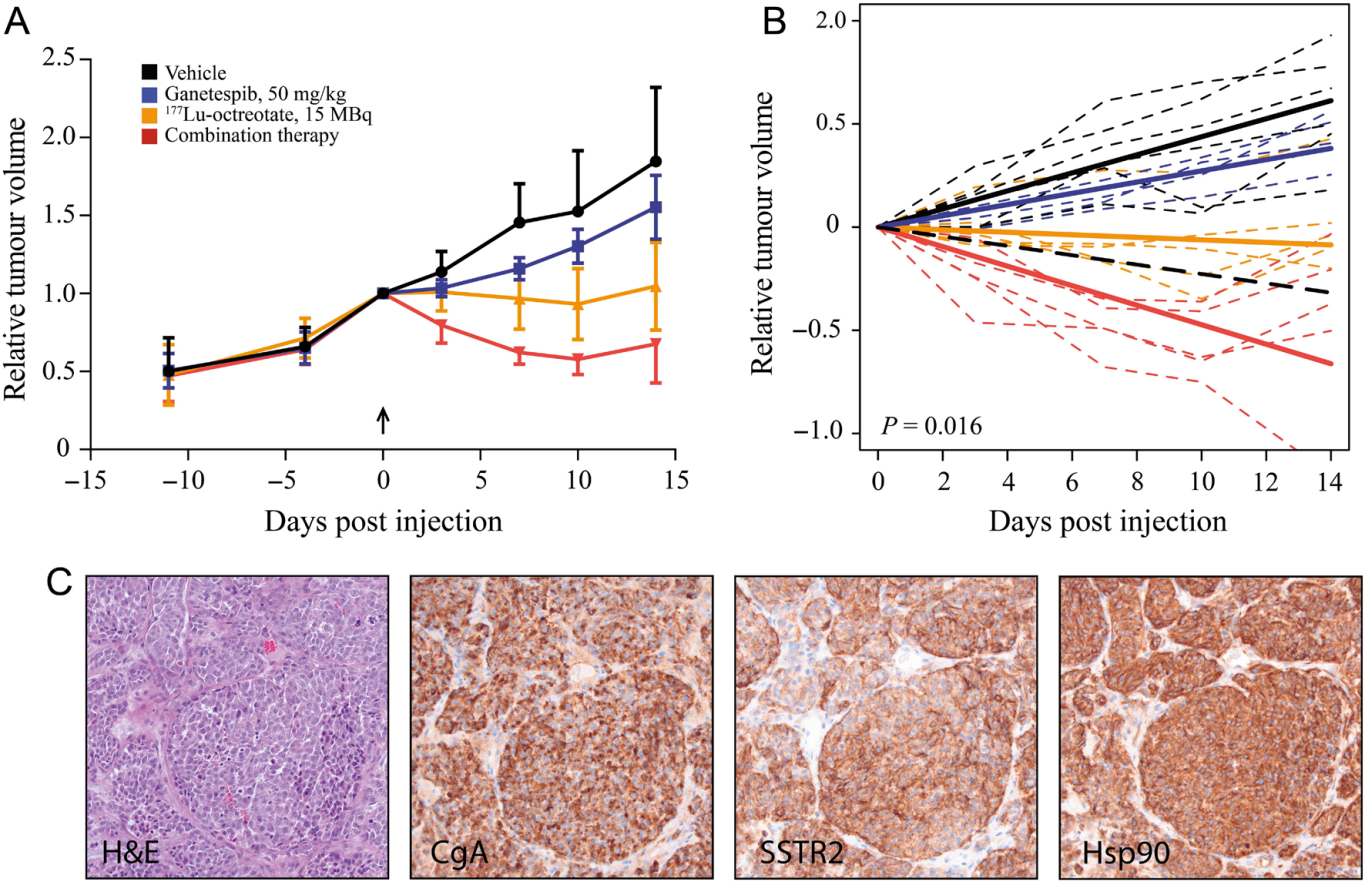
Hsp90 inhibition improves the tumour-killing effect of ^177^Lu-octreotate in the GOT1 xenograft model in a synergistic manner. (A) The combination therapy of Hsp90i ganetespib and ^177^Lu-octreotate had a higher tumour-killing effect than monotherapy. One extreme individual (with a value >3.5 standard deviations (s.d.) from the mean) was excluded from the data. Symbols represent mean and error bars show s.d. The arrow indicates start of treatment. (B) Longitudinal analysis using a linear mixed model applied to time points with treatment effect (days 0 to 10) revealed that ganetespib and ^177^Lu-octreotate synergised to reduce tumour volume. Dashed lines represent individual mice tumours, straight thicker lines represent mean linear regressions for each treatment group and the dashed black straight line represents predicted linear regression of an additive effect. (C) The GOT1 xenograft model had a morphology consistent with a neuroendocrine tumour, including strong expression of neuroendocrine protein markers. The tumours also stained strongly for SSTR2 and Hsp90α/β. CgA, chromogranin A; H&E, haematoxylin and eosin stain; Hsp90, heat shock protein 90; SSTR2, somatostatin receptor subtype 2.

The tumour volume of all mouse tumours were measured twice weekly and the experiment was ended after 14 days (Fig. 2A and Supplementary Fig. 1). Under the experimental conditions chosen for the monotherapies, ganetespib alone did not lead to tumour regression but led to 16% tumour growth inhibition relative to vehicle-treated tumours at the end point of the experiment. ^177^Lu-octreotate led to 7% tumour regression compared with treatment start at the time of maximum response (10 days). The combination therapy had the overall best response, leading to 42% tumour regression compared with treatment start after 10 days. To determine whether there was synergy between ganetespib and ^177^Lu-octreotate in reducing tumour volume, we performed a longitudinal analysis using a linear mixed-model analysis applied over time points with sustained treatment effect (0 to 10 days, Fig. 2B). The analysis indicated that the two treatments synergised in reducing tumour volume, but this was only significant (*P* = 0.016) after excluding one extreme individual in the ganetespib monotherapy group with a value >3.5 standard deviations from the mean (otherwise *P* = 0.13). The extreme individual belonged to the ganetespib-treated group and had a tumour which regressed 48% after 14 days. The other ganetespib-treated mice had tumours that instead progressed, with an average tumour growth of 55%.

We also measured chromogranin A in plasma, a biomarker used to monitor SINETs (Arnold *et al.* 2008, Yao *et al.* 2011), at the end of experiment. Human chromogranin A could be detected in the plasma of all mice with xenografted GOT1 tumours. There was, however, no correlation between chromogranin A levels and tumour volume or treatment modality. We also quantified tumour regression according to Becker (Becker *et al.* 2003) and compared the amount of remaining tumour tissue between vehicle-treated tumours and the other treatment groups (Supplementary Fig. 2). All vehicle-treated tumours had more than 50% tumour remaining (score 3). In the other treatment groups the majority of the tumours also scored 3, but in addition contained one or two tumours with 10–50% remaining tumour (score 2). The difference between vehicle-treated tumours and the other treatment groups or between vehicle-treated tumours and all other treatment groups together was not statistically significant. Morphological analysis of tumour xenografts showed neuroendocrine differentiation with strong immunohistochemical staining for synaptophysin, chromogranin A, serotonin and pan-cytokeratin (Fig. 2C). All xenografted tumours were strongly stained for SSTR2 and Hsp90α/β.

### The anti-tumour effect of external radiation on patient-derived tumour cells was enhanced by ganetespib

To investigate whether the radiosensitisation that was induced by Hsp90 inhibition might apply to SINETs in general or to a specific subgroup, we investigated the effect of external radiation combined with ganetespib on first-passage patient-derived tumour cells that were isolated from patient metastatic tumours collected at surgery. Tumours from eight patients with varying clinical characteristics were used (Table 1). The diagnosis of all patient tumours was re-evaluated and confirmed to be well-differentiated neuroendocrine tumours of grade 1 or 2. All patient tumours were positive for chromogranin A, synaptophysin, serotonin, SSTR2 and Hsp90.

First-passage single-cell suspensions were generated from patient tumours, seeded onto plates and treated after adhering. To verify the authenticity of the cultures generated, cell blocks were prepared and stained for synaptophysin, chromogranin A, serotonin, pan-cytokeratin, SSTR2 and Hsp90. All the protein markers investigated were strongly expressed.

**Figure 3:**
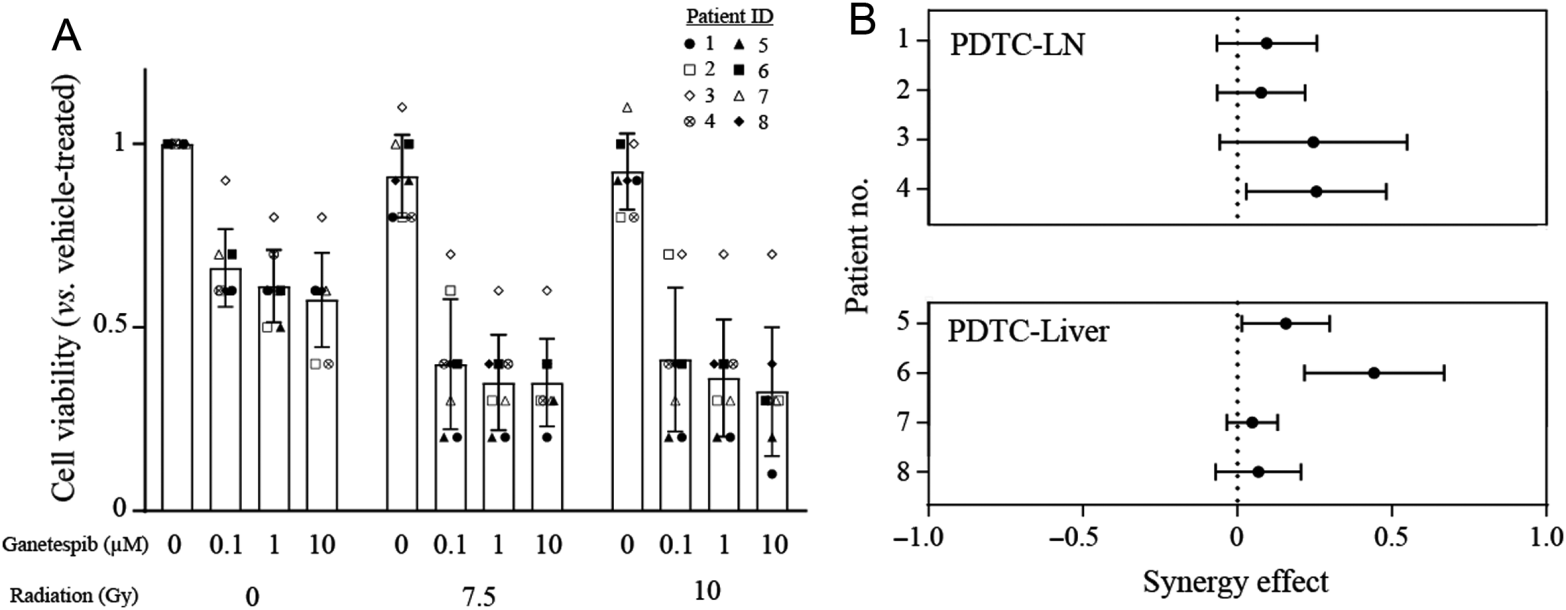
Hsp90i ganetespib synergises with external radiation to reduce the viability of patient-derived tumour cells. (A) Cells from eight patient tumours were treated with different doses of ganetespib, external radiation or both in combination. Each symbol represent patient-derived tumour cells from a specified patient, bars represent mean of all patient-derived tumour cells and error bars denote standard deviation. (B) The measured synergy effect for each patient tumour sample showed that all tumours tended towards synergy. PDTC-LN/PDTC-Liver, patient-derived tumour cells from lymph node metastases/liver metastases.

Tumour cells were treated with ganetespib, external radiation or a combination of both at all dose combinations (Fig. 3A). Cells treated with the combination showed much lower viability compared to the monotherapies. When we performed an analysis of synergy by fitting a linear trend line for the cell viability as a function of the concentration of ganetespib, we could see the viability of radiation-treated cells decrease more quickly, confirming that there was a synergistic interaction (7.5 Gy: *P* = 0.026; 10 Gy: *P* = 0.014) (Supplementary Fig. 3). For all eight individual patient tumours, there was a consistent trend towards synergy (Fig. 3B).

### Hsp90 was overexpressed in neuroendocrine tumours

**Figure 4:**
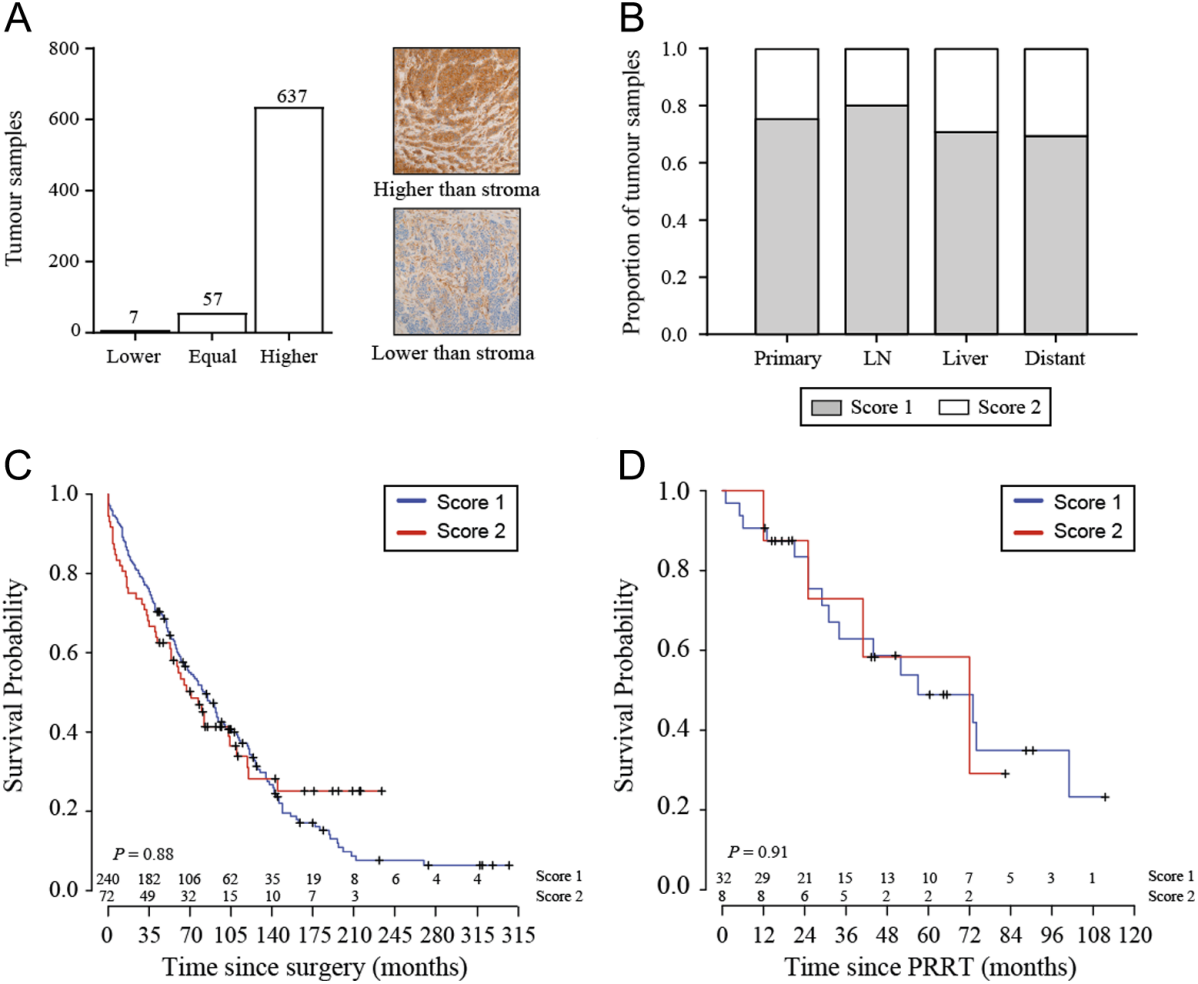
Expression of Hsp90 in SINETs and its relation with patient survival. (A) Most SINETs had higher expression of Hsp90 in the tumour cells compared to the surrounding tumour stroma. (B) The expression level of Hsp90 was scored ‘1’ or ‘2’ based on staining intensity and showed no significant difference in distribution between tumour sites. (C) Kaplan–Meier survival analysis of a SINET cohort with 312 patients showed no association between Hsp90 expression in the patient tumour and patient survival. (D) In a cohort of 43 SINET patients treated with ^177^Lu-octreotate, no association was found between Hsp90 expression and patient survival.

In response to proteotoxic stressors, expression of heat shock proteins is upregulated to enable cell survival. Such stressors are usually prevalent in cancer cells, and Hsp90 inhibition has thus been proposed as a therapeutic opportunity with malignant tumours (Whitesell & Lindquist 2005). The expression of Hsp90 in SINETs has not been reported previously. We therefore examined the expression of Hsp90 in a cohort of 767 SINETs from 379 patients with immunohistochemistry. Hsp90 was found to be highly upregulated in tumour cells relative to the surrounding tumour stroma (Fig. 4A). The Hsp90 expression also varied between tumours, but no differences in expression levels were found by comparing tumour sites (Fig. 4B). Since a correlation has been found between high Hsp90 expression and a poor prognosis in other cancer types (Kang *et al.* 2010, Wang *et al.* 2013, Dimas *et al.* 2018), we investigated whether this was the case also for SINETs. We did not find any relationship between Hsp90α/β expression and overall survival in our cohort, even when we stratified by tumour location (Fig. 4C). Since we found that Hsp90 inhibition sensitised cancer cells to radiation, we also investigated whether innate variations in Hsp90 protein levels could be used to predict treatment benefit from ^177^Lu-octreotate. Among investigated tumours, we identified 43 patients that had gone through ^177^Lu-octreotate therapy and retrospectively investigated the association between Hsp90α/β expression and overall survival after ^177^Lu-octreotate-treatment for these patients. In this cohort, no association between Hsp90α/β expression and patient survival was found (*P* = 0.91; Fig. 4D).

## Discussion

^177^Lu-octreotate therapy is a promising treatment modality for patients with neuroendocrine tumours. The tumour-killing efficacy of ^177^Lu-octreotate therapy is however limited and needs to be improved. In this article we found that the ^177^Lu-octreotate therapy can be enhanced by inhibiting the heat shock response of tumour cells. Hsp90 inhibition as a way of increasing the effect of ^177^Lu-octreotate was identified by means of a large-scale screening for inhibitors that synergise with radiation in SINET cells. By performing a class analysis on all inhibitors in the screening, we found that Hsp90 inhibitors were overrepresented among inhibitors that synergised with radiation in the GOT1 cell line. We also observed that Hsp90 inhibitor ganetespib improved ^177^Lu-octreotate therapy synergistically *in vivo* and that the potentiation of radiation-induced tumour-killing persisted *ex vivo* in SINET patient-derived tumour cells.

Hsp90 is a protein-folding chaperone belonging to the group of heat shock proteins that assist in correct folding of proteins and prevention of faulty protein aggregation. When subjected to stress, cells upregulate expression of Hsp90 to aid in maintaining cellular homeostasis by preventing harmful protein-protein interactions (Whitesell & Lindquist 2005). The Hsp90 chaperone can function both as a potentiator of phenotypic manifestations by assisting oncoproteins and as a capacitator by allowing tumours to tolerate external stress, and also alterations within the cell itself (Karras *et al.* 2017). Thus, it is perhaps not surprising to find expression of heat shock proteins upregulated in several types of human cancers, both solid and haematological (Yufu *et al.* 1992, Chant *et al.* 1995, Ralhan & Kaur 1995, Conroy *et al.* 1998). Here, for the first time we have shown that Hsp90 expression is higher in SINETs relative to tumour stroma. Inhibition of Hsp90 has previously been shown to sensitise tumour cells to ionising radiation in other types of cancer. Hsp90 is involved in a wide range of cellular processes and studies in yeast have identified more than 600 putative Hsp90 substrates (Zhao *et al.* 2005). Finding the mechanism of Hsp90i radiosensitisation is therefore a complex task. Proteomic analysis has however shown that Hsp90 inhibition predominantly targets kinases and components of the DNA damage response (Sharma *et al.* 2012) and in particular inhibition of the DNA damage repair has been reported in several other studies, mainly in the context of delayed or reduced clearance of irradiation-induced γH2AX foci after Hsp90i treatment (Dote *et al.* 2006, Stingl *et al.* 2010, Djuzenova *et al.* 2012, Gandhi *et al.* 2013). Authors have attributed this to the two major DNA double-strand repair mechanisms: homologous recombination repair and non-homologous end joining. As mentioned above, kinases are also sensitive to Hsp90 inhibition, and several kinases that can explain increased sensitivity to radiation are reportedly affected by Hsp90i, including but not limited to EGFR and ERBB2 (Bisht *et al.* 2003, Russell *et al.* 2003). In addition, inhibition of the G2/M checkpoint leading to premature entry or G2/M checkpoint arrest have been suggested mechanisms (Bull *et al.* 2004, Dote *et al.* 2006, Zaidi *et al.* 2012, Wang *et al.* 2016) and it has been speculated that combining Hsp90i and radiation can have beneficial immunogenic anti-tumour effects (Lauber *et al.* 2015). Ganetespib (STA-9090) is a non-geldanamycin, second-generation Hsp90 inhibitor that binds to the ATP-binding pocket of the amino (N) domain and thereby prevents ATP hydrolysis and therefore chaperone function. Ganetespib has shown effect, albeit overall limited, as a monotherapy or in combination with other therapies, in several solid tumour diseases (Goldman *et al.* 2013, Socinski *et al.* 2013, Ramalingam *et al.* 2015, Jhaveri *et al.* 2017, Subramaniam *et al.* 2018). These trials have also demonstrated that ganetespib, in contrast to first-generation Hsp90 inhibitors, has improved solubility and reduced risk of cardiac, ocular and liver toxicities. While Hsp90i to our knowledge has never been studied in the context of ^177^Lu-octreotate therapy, ganetespib combined with radiation has shown promising results in several preclinical studies (He *et al.* 2014, Liu *et al.* 2015, Wang *et al.* 2016), providing support to our hypothesis that ganetespib radiosensitise neuroendocrine tumour cells.

Functional interactions between therapies are often classified into three types: additive (without interaction), antagonistic (a response that is less than additive) or synergistic (a response that is more than additive). When combining treatments, it is advantageous to achieve a synergistic interaction, as this could increase the therapeutic index while reducing toxicity (Fitzgerald *et al.* 2006). This is, however, complicated by the fact that synergistic pairs are rare (4‒10%) (Borisy *et al.* 2003, Zhang *et al.* 2007, Cokol *et al.* 2014), which could explain why previous combination studies that has tried to improve the ^177^Lu-octreotate through combination therapy have not reported synergistic interactions. Here, we performed a comprehensive screening to identify inhibitors that synergised with radiation in GOT1 and P-STS. In our screening, 2‒3% of the inhibitors showed synergistic interactions with radiation and among those only the class of Hsp90 inhibitors were significantly overrepresented. Inhibitors that synergised with radiation represented a diversity of pharmacological principles and differed substantially between GOT1 and P-STS. In particular, Hsp90i, which was highly overrepresented in GOT1, did not show any significant synergy effect in P-STS. The fact that Hsp90i showed significant synergy with radiation and ^177^Lu-octreotate in the GOT1 cell line and the patient-derived tumour cells, but not in the P-STS cell line could be due to several reasons linked to inherent features of this cell line. First, P-STS is not necessarily a good representative of metastatic well-differentiated SINET disease. In fact, P-STS was established from the primary tumour of a grade 3 poorly differentiated neuroendocrine carcinoma (Pfragner *et al.* 2009). GOT1 and *ex vivo* cultures were unlike P-STS established from metastatic grade 1 or 2 well-differentiated neuroendocrine tumours. While likely sharing the same cell of origin, neuroendocrine carcinomas and neuroendocrine tumours differ in several aspects. In terms of genomic background, grade 3 carcinomas frequently harbour *TP53* mutations, which are very rarely found in grade 1 and 2 tumours. *TP53* mutations have been shown to alter tumour cell biology and lead to a worse patient prognosis for patients with neuroendocrine tumours (Sorbye *et al.* 2013). We have previously found that P-STS harbour such a *TP53* mutation (Hofving *et al.* 2018). Patients with poorly differentiated neuroendocrine carcinomas have a worse prognosis, and differ substantially to well-differentiated neuroendocrine tumours in which treatment strategies that are commonly applied. For example, ^177^Lu-octreotate is not approved for the treatment of poorly differentiated neuroendocrine carcinomas, and its efficacy is yet to be proven for these tumours (Strosberg *et al.* 2010). Secondly, P-STS harbour several mutations that are atypical for well-differentiated neuroendocrine tumours and that can influence the radiosensitivity of the cell line, including mutations in *BRCA1* and *BRCA2* (Hofving *et al.* 2018). *BRCA1* and *BRCA2* are essential in DNA double-strand repair and these mutations are not normally observed in SINETs. One of the most commonly described mechanism of HSP90i radiosensitisation is in fact inhibition of DNA double-strand repair (Camphausen & Tofilon 2007). It has been suggested that both *BRCA1* and *BRCA2* are key proteins in the inhibition of DNA double-strand repair by Hsp90 inhibitors (Noguchi *et al.* 2006, Stecklein *et al.* 2012). Thus not only may these aberrant mutations explain the increased radiosensitivity of P-STS cells, but it is also possible that we do not observe a synergistic effect of HSP90i with radiation in P-STS due to the DNA double-strand repair already being impaired.

In conclusion, we have described a novel combination therapy to improve the clinically available ^177^Lu-octreotate therapy for treatment of neuroendocrine tumours. In a synergistic manner, the Hsp90 inhibitor ganetespib improved ^177^Lu-octreotate efficacy in the GOT1 cell line and xenograft model. The radiosensitising effect was also observed in patient-derived tumour cells. We propose that the combination of Hsp90i and ^177^Lu-octreotate should be evaluated in a clinical setting.

## Supplementary Material

Supplementary Fig. 1

Supplementary Fig. 2

Supplementary Fig. 3

Supplementary Table 1

## Declaration of interest

The authors declare that there is no conflict of interest that could be perceived as prejudicing the impartiality of the research reported.

## Funding

This work was financed by grants from the Swedish Research Council, the Swedish Cancer Society, the Assar Gabrielsson Research Foundation, the Wilhelm and Martina Lundgren Foundation for Scientific Research, the Jubilee Clinic Cancer Research Fund, the Sahlgrenska University Hospital Funds, the BioCARE National Strategic Research Programme and the Swedish state under the agreement between the Swedish government and the county councils, the ALF-agreement (ALFGBG-725101/725031).
