# Supplementary figures and images for: 177Lu-octreotate therapy for neuroendocrine tumours is enhanced by Hsp90 inhibition
Source: Endocr Relat Cancer. 2019 Feb 7;26(4):437–49. doi: 10.1530/ERC-18-0509 (PMC6391910; doi:10.1530/ERC-18-0509)

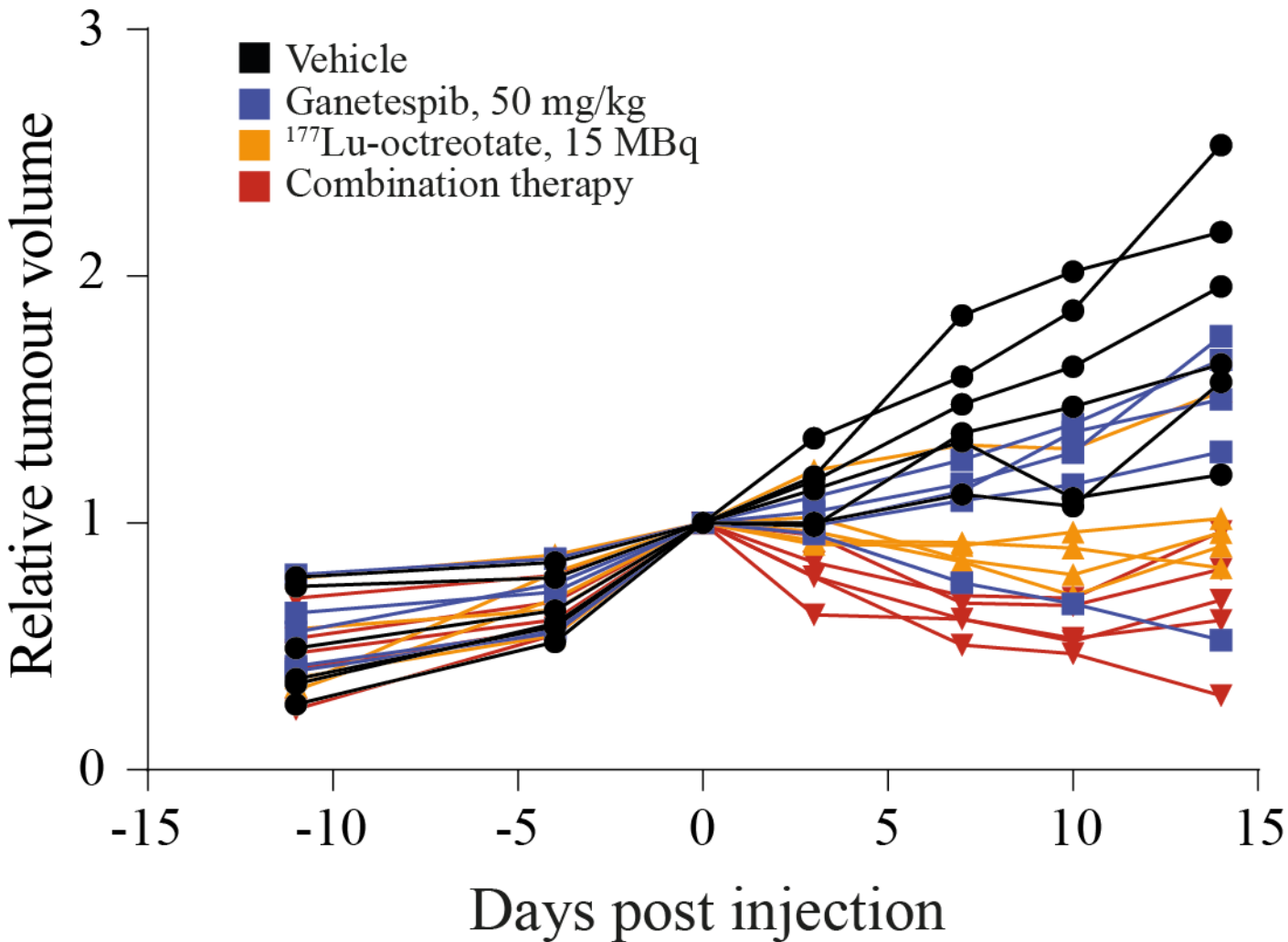

Supplement: Supplementary Fig. 1 [file supplementary_figure_1.pdf]

A

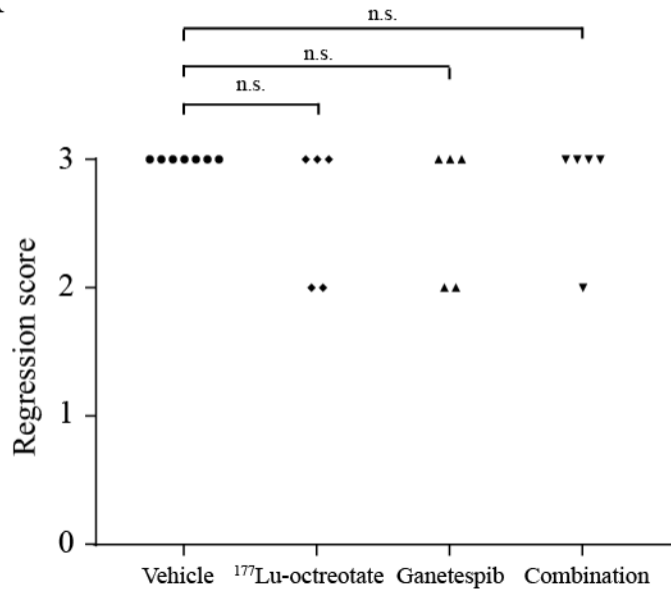

B

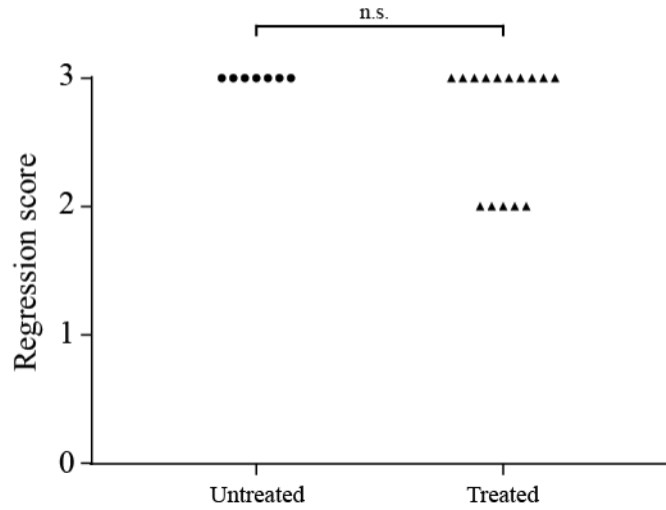

Supplement: Supplementary Fig. 2 [file supplementary_figure_2.pdf]

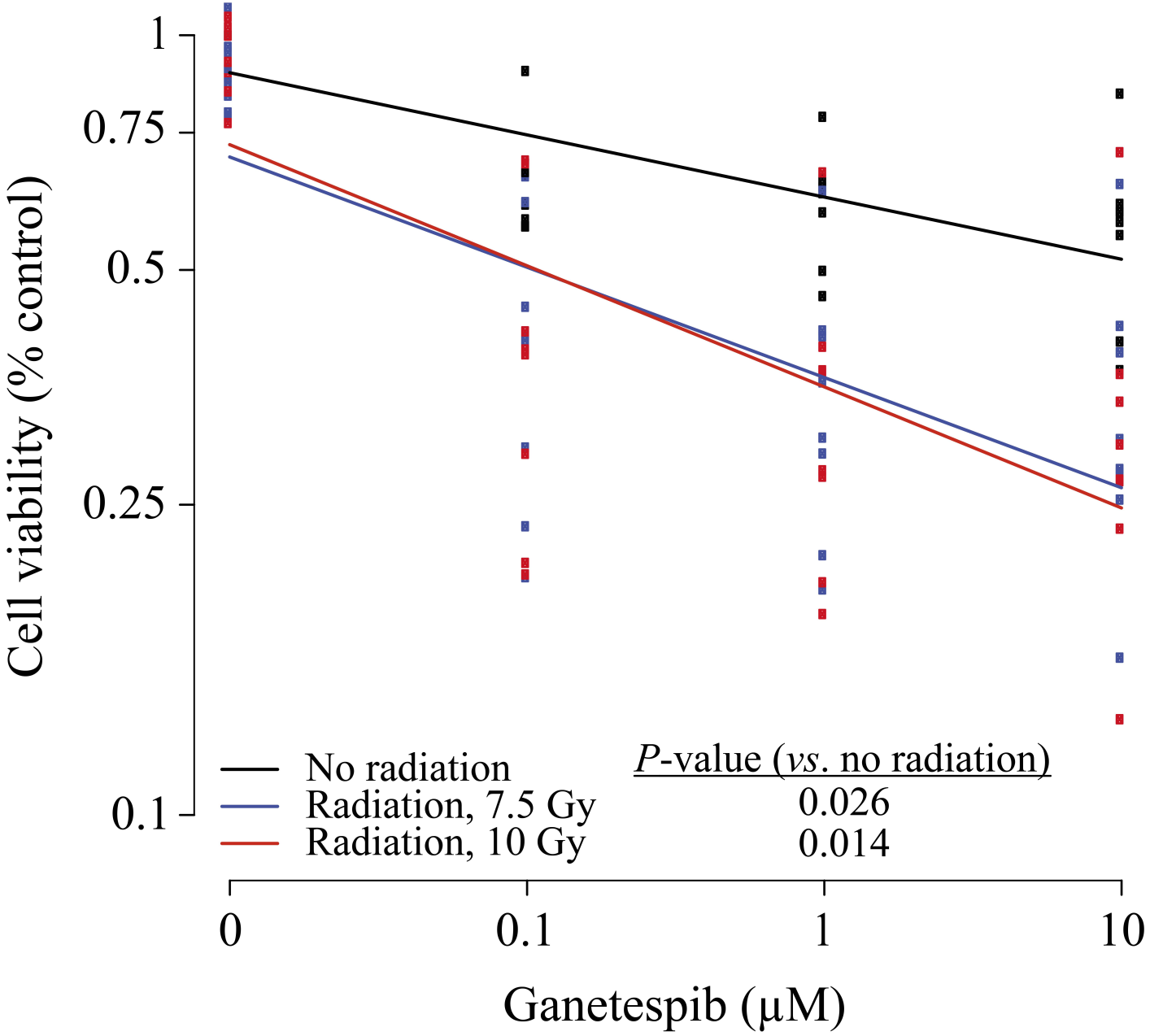

Supplement: Supplementary Fig. 3 [file supplementary_figure_3.pdf]
